# Metal-binding amino acid ligands commonly found in metalloproteins differentially fractionate copper isotopes

**DOI:** 10.1038/s41598-024-52091-7

**Published:** 2024-01-22

**Authors:** Corday R. Selden, Kathrin Schilling, Linda Godfrey, Nathan Yee

**Affiliations:** 1 Department of Marine and Coastal Sciences, Rutgers, University, New Brunswick, NJ USA; 2 Department of Earth and Planetary Sciences, Rutgers University, Piscataway, NJ USA; 3 Department of Environmental Health Sciences, Mailman School of Public Health, Columbia University, New York, NY USA; 4 Department of Environmental Sciences, Rutgers University, New Brunswick, NJ USA

**Keywords:** Metalloproteins, Geochemistry, Metals

## Abstract

Copper (Cu) is a cofactor in numerous key proteins and, thus, an essential element for life. In biological systems, Cu isotope abundances shift with metabolic and homeostatic state. However, the mechanisms underpinning these isotopic shifts remain poorly understood, hampering use of Cu isotopes as biomarkers. Computational predictions suggest that isotope fractionation occurs when proteins bind Cu, with the magnitude of this effect dependent on the identity and arrangement of the coordinating amino acids. This study sought to constrain equilibrium isotope fractionation values for Cu bound by common amino acids at protein metal-binding sites. Free and bound metal ions were separated via Donnan dialysis using a cation-permeable membrane. Isotope ratios of pre- and post-dialysis solutions were measured by MC-ICP-MS following purification. Sulfur ligands (cysteine) preferentially bound the light isotope (^63^Cu) relative to water (Δ^65^Cu_complex-free_ = − 0.48 ± 0.18‰) while oxygen ligands favored the heavy isotope (^65^Cu; + 0.26 ± 0.04‰ for glutamate and + 0.16 ± 0.10‰ for aspartate). Binding by nitrogen ligands (histidine) imparted no isotope effect (− 0.01 ± 0.04‰). This experimental work unequivocally demonstrates that amino acids differentially fractionate Cu isotopes and supports the hypothesis that metalloprotein biosynthesis affects the distribution of transition metal isotopes in biological systems.

## Introduction

Copper (Cu) is an essential micronutrient due to its role as a cofactor in a myriad of key proteins. Cuproproteins drive diverse physiological functions, including respiration (cytochrome *c* oxidase), superoxide scavenging (Cu/zinc superoxide dismutase), methanotrophy (methane monooxygenase) and nitrogen cycling (nitrite reductase, azurin)^1,2^. The binding of Cu in these distinct structures controls their folding and function. The most common Cu-binding ligands in proteins are cysteine and histidine, which both occur in roughly two-thirds of experimentally-resolved Cu-binding sites^1^. Cysteine coordinates Cu with the sulfur (S) atom of a thiol while histidine ligates Cu with a nitrogen (N) atom in its imidazole ring. Oxygen (O) ligands are comparatively less common but still prevalent: glutamate and aspartate, which bind Cu with O atoms in their carboxyl side chains, occur in 7% and 6% of Cu-binding sites, respectively. Bonds between Cu and these ligands (S, N, O) differ in their intramolecular vibrational frequencies and, consequently, their energy, strength, and stability^3–5^.

Advances in mass spectrometry^6,7^ have facilitated the use of Cu isotopes as tracers of biological function^3,8–30^. Copper has two stable isotopes with masses of 63 and 65 amu, respectively. In animals, these isotopes are fractionated among different tissue and fluid types^3^. Because fractionation patterns differ with disease state^3^, Cu isotope ratios has been proposed as diagnostic indicators of Alzheimer’s disease^20,21^, breast, ovarian and colorectal cancer^27,28^ as well as multiple diseases of the liver^9,26, 29, 31^. However, development of such diagnostics is presently hampered by numerous confounding variables including organisms’ sex^8,10^, age^13,19^, diet^32^, and menopausal state^15,33^.

Developing robust biomarkers for applications in human health, among other potential applications in the geosciences, requires a clear model of the mechanisms which drive biological Cu isotope fractionation. Previous studies have demonstrated significant variations in Cu isotope ratios in cuproproteins, which are hypothesized to be caused by differences in Cu binding site configurations^17,34^. Ab initio calculations based on density functional theory (DFT) predict that the identity and geometry of the coordinating amino acids at protein Cu-binding sites determine their relative affinity for ^63^Cu versus ^65^Cu^4^. Accordingly, the differential expression of structurally-distinct proteins would influence the isotopic composition of a cell or tissue. While numerous studies have relied on ab initio estimates to explain variations in Cu isotope ratios measured in biological samples^8,9, 27^, there is currently no experimental data to test or validate these theoretical predictions.

In this study, Cu^2+^ isotope fractionation by amino acid ligation was quantified in laboratory experiments using Donnan dialysis to separate bound versus free Cu^2+^ and multicollector inductively coupled plasma mass spectrometry (MC-ICP-MS) to measure isotope abundance. We conducted experiments with the metal-binding amino acids cysteine, histidine, glutamate, and aspartate. Our objective was to experimentally determine the (mass-dependent) isotope effects associated with Cu^2+^ ligation by these amino acids and to provide direct evidence that distinct protein metal-binding sites differentially fractionate Cu isotopes. The results of this work offer insight into the biomolecular mechanisms which underpin Cu isotope fractionation among proteins and at higher levels of biological systems.

## Materials and methods

### Chemical reagents

All reagents were prepared using ultrapure deionized water (18.2 MΩ·cm, Milli-Q, Millipore). High purity 4-morphlineethanesulfonic (MES) hydrate (BioUltra, Sigma-Aldrich) was used to buffer reactions. Trace analysis quality (> 99.995%) strontium nitrate (Sr(NO_3_)_2_) was used as a background electrolyte^35,36^. Ligands were reagent-grade (> 95%) or higher: l-cysteine (reagent grade, Sigma-Aldrich), l-histidine (BioUltra [< 99.5%], Sigma-Aldrich), l-aspartic acid (reagent grade, Sigma-Aldrich), and l-glutamic acid (reagent grade, Sigma-Aldrich). A Cu nitrate standard (Sigma-Aldrich) was used as the Cu source in all experiments. All solutions were prepared and stored in metal-free centrifuge tubes (Labcon), which were soaked for 2 days in 20% nitric acid (HNO_3_) and rinsed with ultrapure water prior to use. Following dialysis, samples for Cu analysis were stored in clean 2 mL microcentrifuge tubes (2 days, 20% HNO_3_) at 4 °C to minimize evaporation.

### Cu ligation experiments

Copper stock solutions (100 µM) contained 10 mM Sr(NO_3_)_2_ as a background electrolyte and 5 mM MES hydrate as a buffer, and were adjusted to pH 6.0 using 1 M sodium hydroxide (NaOH). Copper solutions were mixed with either cysteine, histidine, aspartate, or glutamate and allowed to equilibrate overnight (≥ 16 h) under ambient laboratory conditions. All Cu ligation experiments were conducted with 100 µM amino acids except cysteine. A ratio of 100 µM Cu and 50 µM cysteine was used in cysteine experiments to avoid quantitative Cu binding. Limiting disulfide bridging, the cysteine-Cu solution was made under anoxic conditions using N_2_-purged ultrapure water. Previous X-ray absorption near edge structure (XANES) analyses have shown that when Cu^2+^ is reacted with cysteine, the oxidation state of Cu remains as 2+^37^.

After the complexation reaction was complete, free Cu^2+^ was separated from amino acid-bound Cu^2+^ via an equilibrium Donnan dialysis procedure adapted from Nolan et al.^38^ and Ryan et al.^36^. Briefly, two solutions were added to opposing 1.5 ml cells in Teflon micro-equilibrium dialyzers (Harvard Apparatus): (1) a Cu solution containing dissolved amino acids, and (2) an acceptor solution containing 10 mM Sr(NO_3_)_2_ and 5 mM MES hydrate, adjusted to pH 6.0. These cells were separated by a strong-acid cation-exchange membrane (Nafion-117, E.I. Dupont de Nemours) which permits passage of free but not bound Cu^2+^. Free Cu^2+^ thus equilibrates across the membrane and accumulates in the acceptor solution, while bound Cu is retained in the donor solution.

Before dialysis, Teflon dialyzers were soaked overnight in 2% HNO_3_ and thoroughly rinsed with ultrapure deionized water. Membranes were rinsed and stored in ultrapure deionized water for a minimum of 2 days. Immediately prior to use, membranes were pre-conditioned by two successive soaks in 10 mM Sr(NO_3_)_2_ for a minimum of 2 h each. The dialysis system was then assembled and, as a final pre-conditioning step, filled with acceptor solution adjusted to pH 6 for an additional 2 h. This solution was then removed and Cu separation was performed by adding the Cu^2+^ donor solution and fresh acceptor solution to the opposing cell in the dialyzer. Dialysis was carried out for approximately 27 h. No-ligand control experiments indicated that dialysis reached mass and isotopic equilibrium within 1 day (Suppl. Fig. 1; Table 1). Positive control experiments performed with the amino acid glycine, which has no side chain, confirmed that Cu-binding by carboxyl and amine terminal groups was negligible under the reaction conditions (Suppl. Fig. 2). At the end of each experiment, samples of initial (t = 0) and final (t = f) acceptor and donor solutions were collected for analysis. All experiments were replicated and no-ligand controls were run in parallel to verify that equilibrium was reached and that Cu isotope fractionation did not occur without amino acid complexation. Experimental trials with cysteine (each with one control and three replicate experiments) were carried out on two separate occasions to confirm that the results were reproducible.

**Table 1:** Equilibrium Cu concentrations and δ^65^Cu^a^ values after (27 ± 1 h) dialysis of no-ligand control i.e., donor solution containing 100% free Cu (mean ± 2 SE, n = 5)^b^.

Solution	Initial Cu (µg)	Initial δ^65^Cu (‰)	Final Cu (µg)	Final δ^65^Cu (‰)
Donor	10.7 ± 1.4	0.88 ± 0.07	3.8 ± 0.4	0.89 ± 0.03
Acceptor	Undetectable	N/A	3.3 ± 0.4	0.90 ± 0.04
Total	10.7 ± 1.4	0.88 ± 0.07	7.1 ± 0.9	0.90 ± 0.08^c^

^a^Calculated as in Eq. (1).

^b^Control experiments were conducted alongside each set of amino acid experiments; control replicates were thus performed on different days, each with freshly-made acceptor and Cu donor solutions.

^c^Calculated value based on mass balance of final donor and acceptor δ^65^Cu values; error is propagated.

Copper concentrations were determined for acceptor, donor and no-ligand control solutions collected before (t = 0) and after (t = f) Donnan dialysis using inductively coupled plasma optical emission spectrometry (ICP-OES). Aliquots were acidified with 2% HNO_3_ and analyzed on an iCAP 7400 ICP-OES (ThermoFisher) at a wavelength of 325 nm. Detection limits were assessed daily and ranged from 0.12 to 1.10 µM Cu (3σ, n > 5 blanks). The mean Cu recovery in control experiments was 66.7 ± 1.7%. We attribute this loss of Cu from solution within the dialysis system to adsorption onto the membrane^39^. Adsorption of free Cu^2+^ on the membrane did not, however, fractionate Cu isotopes; the isotopic composition of the residual dissolved Cu (δ^65^Cu =  + 0.90 ± 0.08‰) was within error of its initial Cu isotope value (+ 0.88 ± 0.07‰; Table 1). Copper recovery was greater in experiments with cysteine, glutamate and aspartate; however, only half of Cu was recovered during trials with histidine (Suppl. Table 1), which we attribute to partial adsorption of histidine-Cu complexes on the membrane.

### Cu purification and isotope analysis

Sample purification and preparation for isotope analysis was performed in a clean lab under positive pressure inside laminar-flow clean benches, using ultrapure deionized water (Milli-Q, Millipore) and high-purity acids (Aristar Ultra, BDH). Copper samples were separated using AG MP-1 anion resin (100–200 µm mesh, BioRad) in PolyPrep columns (BioRad). Samples were stored and the Cu fraction collected in Teflon beakers, which were cleaned by soaking in aqua regia (3 parts HCl to 1 part HNO_3_; 2 days) followed by hot 50% HNO_3_ and 10% hydrofluoric acid (HF; 2 days). Resin was discarded after a single use, and the emptied columns were stored in 20% hydrochloric acid (HCl).

Solution aliquots containing 15 µg Cu were transferred to acid-washed Teflon beakers and dried. Subsequently, 100 µl concentrated hydrogen peroxide (H_2_O_2_) was added and samples were refluxed for 15 min at ~ 150 °C to remove organics, then again evaporated to dryness and finally dissolved in 250 µl 6 M HCl. Copper was separated from the sample matrix following Borrok et al.^7^. Briefly, PolyPrep columns (2 ml stem, 10 ml reservoir) were loaded with 1.5 ml of resin. Resin beds were further rinsed with 10 ml of ultrapure deionized water and conditioned using 3 ml 6 N HCl. Samples were then loaded on the columns and washed twice with 0.5 ml 6 N HCl then once with 3 ml 6 N HCl to ensure complete removal of Sr and sodium. Copper was subsequently eluted in 29 ml 6 N HCl and placed on a hotplate to evaporate samples to complete dryness.

Copper samples were converted to nitrate salts via addition of a few drops of concentrated HNO_3_, dried, then redissolved in 2% HNO_3_ with 0.05% HF. Sample dilutions were adjusted to match the concentration (200 µg/L Cu) of the bracketing Cu standard and analyzed on a multicollector inductively coupled plasma mass spectrometer (MC-ICP-MS; ThermoScientific Neptune Plus). A certified Cu wire reference material (HICU-1; National Research Council of Canada) was used for standard bracketing at two sample intervals^40^. Instrument accuracy was assessed by measuring an 1838 Cu penny standard and the Cu source solution for the experiments (Suppl. Table 2). All values were corrected by the mean difference between HICU-1 and NIST SRM 976, which was measured intermittently throughout every run. Copper isotopic composition is reported relative to the NIST SRM 976 standard:

1 $$\delta {}^{65}{\text{Cu}} (\textperthousand)=\left[\frac{{\frac{{}^{65}{\text{Cu}}}{{}^{63}{\text{Cu}}}}_{sample}}{{\frac{{}^{65}{\text{Cu}}}{{}^{63}{\text{Cu}}}}_{SRM976}}-1\right]\times 1000$$

Contamination was assessed by examining Cu recovered from ultrapure water and acceptor solutions (1 ml), which were subjected to column chemistry and resuspended in 1 ml dilute HNO_3_. Copper concentrations in these solutions averaged 2.4 (n = 3) and 4.7 (n = 4) ng ml^−1^, respectively. Copper background from reagent impurities, sample handling and the environment did not exceed 0.5% of total Cu in our samples. Recovery and fractionation following column chemistry was assessed using column-cleaned initial (t = 0) Cu donor solutions, each containing a different amino acid and made on a different day. Average Cu recovery for these solutions was 98 ± 5% (n = 7 columns). The mean $\updelta$^65^Cu of these solutions was + 0.88 ± 0.07‰ (n = 5 solutions, which were each individually analyzed ≥ 3 times) following column chemistry; this value is within error of the mean $\updelta$^65^Cu of the Cu stock solution, which was + 0.89 ± 0.02‰ across all instrument runs (n = 20; Suppl. Table 2). From this observation, we conclude that reagent impurities had a negligible effect on our experimental results.

### Cu mass balance

The δ^65^Cu value of complexed Cu was calculated following a mass balance equation derived from Ryan et al.^36^:

2 $${\updelta{}^{65}{\text{Cu}}}_{complexed}=\frac{{\updelta{}^{65}{\text{Cu}}}_{total}-({\updelta{}^{65}{\text{Cu}}}_{free}\times {f}_{free})}{(1-{f}_{free})}$$

where δ^65^Cu_free_ indicates the Cu isotope abundance measured directly in the acceptor solution at t = f (also referred to here as ${\updelta{}^{65}Cu}_{acceptor}$), and *f*_free_ indicates the relative proportion of free Cu within the system, calculated as:

3 $${f}_{free}=\frac{{2 \times mass}_{acceptor}}{({mass}_{donor}+{mass}_{acceptor})}$$

The relative isotope abundance of all dissolved Cu (δ^65^Cu_total_) was calculated as follows:

4 $${\updelta{}^{65}{\text{Cu}}}_{total}={\updelta{}^{65}{\text{Cu}}}_{acceptor} \times {f}_{acceptor}+{\updelta{}^{65}{\text{Cu}}}_{donor} \times {f}_{donor}$$

where δ^65^Cu_total_ represents the δ^65^Cu of the total dissolved pool following dialysis. ${\updelta{}^{65}Cu}_{acceptor}$ (equal to ${\updelta{}^{65}{\text{Cu}}}_{free}$ above) and ${\updelta{}^{65}{\text{Cu}}}_{donor}$ indicate the Cu isotope abundance at t = f in the acceptor and donor solutions, respectively, and $\text{f}$ indicates the fractional contribution of each chamber to the sum Cu mass at t = f:

5 $${f}_{donor}=\frac{{mass}_{donor}}{({mass}_{donor}+{mass}_{acceptor})}$$

Finally, Cu isotope separation values between complexed and free Cu were calculated as follows:

6 $$\Delta {{\text{Cu}}}_{complexed-free}={\delta {}^{65}{\text{Cu}}}_{complexed}-{\delta {}^{65}{\text{Cu}}}_{free}$$

## Results and discussion

### Cu binding

Reaction of Cu with amino acids resulted in metal ligation and the retention of ligand-bound Cu in the chamber of the dialyzer which contained amino acids. Copper retention was greatest for cysteine (83.4 ± 04% [n = 3] and 78.5 ± 3.0% [n = 3] of total dissolved Cu^2+^ in two separate trials), lower for the carboxylate ligands (67.0 ± 0.6% [n = 3] for glutamate, 66.2 ± 0.8% [n = 3] for aspartate), and 57.6 ± 0.8% [n = 2] for histidine (Suppl. Table 1). Free Cu^2+^ migrated across the dialysis membrane and was recovered in acceptor solutions (Suppl. Fig. 1). Free Cu^2+^ was distributed equally between the donor and acceptor solutions at mass equilibrium, which was reached in approximately 24 h (Suppl. Fig. 1). Using mass balance equations (1-*f*_free_, Eq. (3); Table 2), we calculated that the proportion of ligand-bound Cu was 57.1 ± 6.0% and 66.9 ± 0.8% for the two cysteine trials, respectively, 34.1 ± 1.1% for glutamate, 32.4 ± 1.6% for aspartate, and 15.3 ± 1.6% for histidine. These values correspond to partition coefficients ([Cu]_free_/[Cu]_bound_) of 0.76 ± 0.19 and 0.50 ± 0.02 for the two cysteine trials, respectively, 1.94 ± 0.10 for glutamate, 2.10 ± 0.15 for aspartate, and 5.58 ± 0.70 for histidine (Suppl. Table 4).

**Table 2:** Cu fractionation induced by amino acids (mean ± 2 SE).

Ligand	Dominant ligand	Amino acid:Cu ratio^a^	# Replicate experiments	*f*_free_ (%)	δ^65^Cu_free_ (‰)	δ^65^Cu_complex_ (calculated, ‰)	Δ^65^Cu_complex-free_ (calculated, ‰)
Cysteine	S	1:2	6	38.0 ± 5.2	+ 1.38 ± 0.08	+ 0.89 ± 0.14	− 0.48 ± 0.18
Histidine	N	1:1	2	84.0 ± 1.6	+ 0.79 ± 0.07	+ 0.78 ± 0.03	− 0.01 ± 0.04
Glutamate	O	1:1	3	65.9 ± 1.1	+ 0.81 ± 0.04	+ 1.07 ± 0.06	+ 0.26 ± 0.04
Aspartate	O	1:1	3	67.6 ± 1.6	+ 0.89 ± 0.03	+ 1.05 ± 0.07	+ 0.16 ± 0.10

^a^Approximate ratio of amino acids to Cu added to experimental donor solutions.

### Cu isotope fractionation

**Figure 1:**
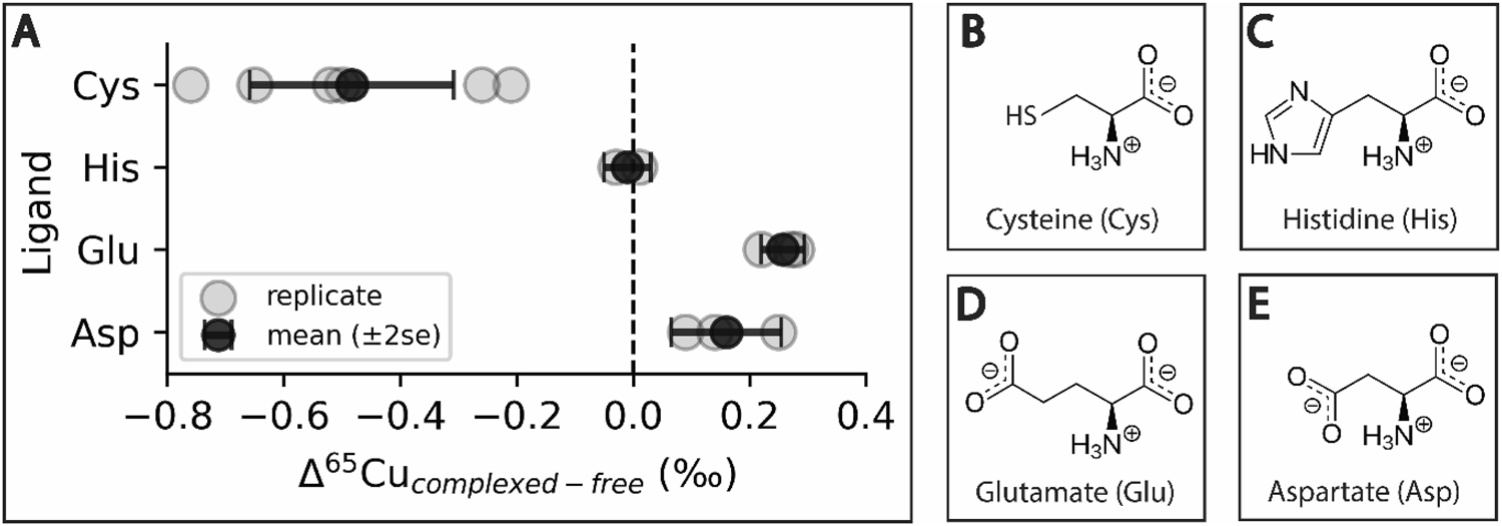
(**A**) Cu isotope fractionation ($\Delta$^65^Cu = $\delta$^65^Cu_bound_ − $\delta$^65^Cu_free_) due to ligation by cysteine (Cys), histidine (His), glutamate (Glu) and aspartate (Asp). Gray points represent $\Delta$^65^Cu calculated for independent experiments; black points represent the mean $\Delta$^65^Cu of replicates (± 2SE). (**B**–**E**) Molecular structures of the amino acids investigated in this study.

Copper complexation by cysteine, glutamate, and aspartate resulted in Cu isotope fractionation (Table 2; Fig. 1). For cysteine, mean (± 2SE) δ^65^Cu_free_ (measured in acceptor solutions at t = f) exceeded mean δ^65^Cu_complexed_ (calculated as in Eq. 2) across all experiments (n = 6), with values of + 1.38 ± 0.08‰ and + 0.89 ± 0.14‰, respectively. For the carboxylates, the opposite trend was observed: δ^65^Cu_free_ was + 0.81 ± 0.04‰ (n = 3) in glutamate experiments and + 0.89 ± 0.03‰ (n = 3) in aspartate experiments while corresponding δ^65^Cu_complexed_ values were + 1.07 ± 0.06‰ (n = 3) and + 1.05 ± 0.07‰ (n = 3). In histidine experiments, δ^65^Cu_free_ and δ^65^Cu_complexed_ were within error, with values of + 0.79 ± 0.08‰ (n = 2) and + 0.75 ± 0.05‰ (n = 2), respectively.

These experimental results indicate that thiol-bearing cysteine preferentially ligated ^63^Cu (Δ^65^Cu_complex-free_ = − 0.48 ± 0.18‰), while glutamate and aspartate, with their carboxyl (O) side chains, favored ^65^Cu (Δ^65^Cu_complex-free_ =  + 0.26 ± 0.04 and + 0.16 ± 0.10‰, respectively; Table 2; Fig. 1). Cooper complexation by N-bearing histidine, in contrast, resulted in negligible isotopic fractionation (Δ^65^Cu_complex-free_ = − 0.01 ± 0.04‰). Copper concentrations and δ^65^Cu for all samples are available in Suppl. Table 3 and averaged results are available in Suppl. Table 4. We note that the large error associated with cysteine ligation was not driven by variability among replicates in the proportion of Cu bound (see above; Table 2), but by variability in the Cu isotope abundances measured in donor and acceptor solutions across replicate experiments (see Suppl. Table 3).

### Comparison with theoretical calculations

Our experimentally-derived Cu isotope fractionation effects are consistent in directionality with mass-dependent equilibrium isotope fractionation. Mass-dependent isotope fractionation occurs because chemical bonds differ in their potential energy as a function of atomic position (i.e., in their potential energy surface). When small changes in atomic position lead to significant changes in potential energy, the bond may be said to have a “steeper” potential energy surface. Heavier isotopes concentrate in the “steeper” (more stable) chemical phase to minimize the overall energy of the system^41^. Bonds with higher natural frequencies of fundamental vibration are associated with steeper potential energy surfaces (i.e., deeper potential energy wells)^41^ and thus have higher spring constants, meaning that they are more resistant to deformation^5,42^. These “stiff” bonds are short and strong—properties associated with lighter ligands, lower coordination numbers, and higher oxidation states^41^. In proteins, stiff bonds are associated with ‘hard’ Lewis bases i.e., those whose electron clouds are less easily distorted under an electric field^43,44^. Carboxyl groups (RO^−^) and water (H_2_O) are hard bases; imidazole is classified as ‘borderline’; thiols (RSH) are ‘soft’ or easily polarized bases^45^. Therefore, when Cu is in the 2+ oxidation state (and a borderline Lewis acid), the carboxylates (glutamate and aspartate) should evince greater preference for ^65^Cu than histidine, while cysteine should accumulate ^63^Cu. This pattern is clearly manifest in our experimental results (Table 2; Fig. 1).

Though consistent in directionality, experimental evidence indicates that the magnitude of Cu isotope fractionation by cysteine is less than that predicted ab initio from DFT^4^. Theoretical calculations from Fujii et al.^4^ predict that, relative to H_2_O, Cu complexation by cysteine should fractionate the Cu isotope pool by − 1.18‰ (Δ^65^Cu_complex-free_) at 25 °C. Our experimentally determined Δ^65^Cu_complex-free_ of − 0.48 ± 0.18‰ is in keeping with the directionally of this value. Both theory and experiment indicate cysteine favors ^63^Cu; however, our measured value is less than half the predicted DFT value. This suggests that ab initio calculations may overestimate isotope fractionation due to ligation by cysteine. This discrepancy between theory and experiment can be explained by differences in the coordination geometry assumed in the DFT model compared to the Cu-amino acid complexation that occurs in real solutions. In the DFT calculations, Fujii et al.^4^ assumed five-fold coordination of Cu^2+^ bonded to H_2_O molecules and a single cysteine ligand. Conversely, extended X-ray absorption fine structure (EXAFS) spectroscopic measurements of Cu^2+^-cysteine complexes in aqueous solutions show Cu^2+^ coordination with three sulfur atoms in the first shell at approximately 2.28 Å^37^. Stiffer bonds and differences in coordination numbers in the Cu^2+^-cysteine that actually form in experimental systems would be expected to mute the predicted Cu isotope fractionation effect.

We have ruled out redox chemistry as a major cause of Cu isotope fractionation by cysteine complexation in our experiments. Previous XANES studies^37^ have shown that the oxidation state of Cu remains as Cu^2+^ upon binding with cysteine. Cu^2+^ reaction with cysteine does not appear to produce Cu^1+^-cysteine complexes, which can be distinguished from EXAFS spectra^37^. Because previous XANES and EXAFS analyses indicate that Cu^2+^ is not chemically reduced to Cu^1+^ by cysteine, we attribute our measured Cu isotope fractionation to complexation to the thiol ligand rather than a redox-driven process.

In our experiments, we observed that both glutamate and aspartate favored ^65^Cu with Δ^65^Cu_complex-free_ values of + 0.26 ± 0.04 and + 0.16 ± 0.10‰ (Table 2; Fig. 1), respectively which differ from the DFT derived value for glutamate of − 0.11‰ relative to H_2_O^4^. These results suggests that DFT models underestimated fractionation due to ligation by carboxylates. As with cysteine, this difference is likely attributable to variability in the coordination geometry and potentially speciation of experimental complexes relative to the simplified models used in DFT calculations. We note that our experimentally determined values are broadly consistent with observation of other organic ligands with O-binding sites. Using a similar dialysis approach, Ryan et al.^36^ reported Δ^65^Cu_complex-free_ values for O-rich ligands ranging from + 0.14 ± 0.11‰ (Suwanee River fulvic acid) to + 0.84 ± 0.30‰ (desferrioxamine B—a siderophore).

For histidine, our experimental results (Table 2; Fig. 1) agreed with the ab initio calculations^4^. These independent approaches both indicate that negligible isotopic fractionation occurs when Cu is bound to histidine under ambient conditions. Ab initio calculations^4^ indicate that Δ^65^Cu_complex-free_ for the aqueous Cu-histidine complexes Cu(His)(H_2_O)_4_^2+^ and Cu(His)(H_2_O)_3_^2+^ should yield Δ^65^Cu_complex-free_ values of − 0.05‰ and − 0.08‰, respectively. Our measured value of − 0.01 ± 0.04‰ is close to these values.

### Comparison with in vivo measurements

Though our measured magnitude of Cu isotope fractionation for cysteine was smaller than predicted by DFT, our data are in excellent agreement with Cu isotope ratios measured in proteins (Table 3). The Cu isotope composition of two structurally-distinct proteins, metallothionein and Cu,zinc (Zn) superoxide dismutase, have previously been measured in vivo^17,34^. Metallothionein binds clusters of metal ions—as many as seven bivalent or 20 monovalent ions—using predominantly the S residues of cysteines^46^. Cu,Zn superoxide dismutase, in contrast, binds only two Cu atoms in identical sites using N from histidine in a tetrahedral configuration^47^. In human cortical tissue, metallothionein Cu is isotopically light (δ^65^Cu = − 0.20 ± 0.21‰) while Cu in Cu,Zn superoxide dismutase is isotopically heavy (δ^65^Cu =  + 0.41 ± 0.27‰)^17^. This pattern also holds true for the yeast *Saccharomyces*, with δ^65^Cu values for metallothionein and Cu,Zn superoxide dismutase of − 2.13‰ and − 1.60‰, respectively^34^. With a difference of − 0.61 ± 0.34‰ (n = 5 subjects) in humans^17^ and − 0.53‰ in yeast^34^, the consistency in the relative partitioning of Cu isotopes between these proteins across biological systems is striking (note: The absolute isotopic value of the two protein pools differs across organisms and individuals due to variability in the dietary source of ﻿Cu^48^, among other factors, and are thus not comparable.) Moreover, the difference between Δ^65^Cu_complex-free_ of cysteine and histidine in our experiments, − 0.47 ± 0.18‰, is within error of these values. The coherence between our in vitro Cu isotope separation value and in vivo protein data suggest that the identity of ligating residues is a major factor dictating metal fractionation in proteins. However, as this study focused exclusively on Cu^2+^, we cannot exclude the potential contribution of oxidation state in accounting for differences among proteins.

**Table 3:** Cu isotope compositions of sulfur (S) and nitrogen (N) bonding environments across levels of biological complexity.

Biocomplexity level	Source	S-rich bonding environment	N-rich bonding environment	Δ^65^Cu_S-N_ (‰)^a^	Reference
Amino acids	Experimental	Cysteine	Histidine	− 0.47 ± 0.18^b^	This study
Amino acids	Theoretical	Cysteine	Histidine	− 1.13	Fujii et al.^4^
Proteins	Human cortices (healthy and Alzheimer’s)	Metallothionein	Cu,Zn superoxide dismutase	− 0.61 ± 0.34^b^	Larner et al.^17^
Proteins	*Saccharomyces*	Metallothionein	Cu,Zn superoxide dismutase	− 0.53	Zhu et al.^34^
Tissues	Humans (healthy)	Blood serum	Red blood cells	− 0.82 ± 0.64^b^	Albarède et al.^8^

^a^For amino acids (experimental and theoretical), this value is the difference between Δ^65^Cu_cysteine-free_ and Δ^65^Cu_histidine-free_, where ‘free’ refers to ions in water.

^b^Error propagated from two times the standard error of individual samples (experiments or subjects).

Isotope partitioning among proteins with S-rich and N-rich binding sites for Cu also manifests at the cellular level consistent with our experimental results: Cells and fluids enriched in ^63^Cu are associated with Cu proteins bearing S-rich binding sites. In healthy humans, for example, blood serum is enriched in ^63^Cu (δ^65^Cu = − 0.26 ± 0.40‰, n = 49) relative to erythrocytes (red blood cells; δ^65^Cu =  + 0.56 ± 0.50‰, n = 49)^8^. Calculations based on DFT support the hypothesis that this fractionation occurs because Cu is ferried to ceruloplasmin—the major Cu protein in blood—via an antioxidant protein (ATOX) and a chaperone protein (ATP7B). The isotope effect is expressed when ATOX and ATP7B bind Cu using two cysteine thiols in a linear configuration^49^. Because the transfer of Cu from ATOX/ATP7B to ceruloplasmin is quantitative, the isotopic composition of Cu in the intermediaries is transferred to ceruloplasmin. Thus, ^63^Cu tends to accumulate in serum while red blood cells accumulate ^65^Cu^8,49^. Albarede et al.^8^ also noted that Cu,Zn superoxide dismutase is more prevalent in red blood cells. The difference in δ^65^Cu between serum and red blood cells of − 0.82 ± 0.49‰ (Table 3), is larger but still within error of the difference we observed between Δ^65^Cu_complex-free_ values for cysteine and histidine (− 0.47 ± 0.18‰; Table 3).

### Medical and environmental applications

As structurally-distinct Cu-binding sites fractionate Cu isotopes among biomolecules (Table 3), patterns in the distribution of Cu isotopes among cells, groups of cells, and their growth environment represent potential biomarkers of cellular activity. For example, like many Cu proteins, both metallothionein (S ligands) and Cu,Zn superoxide dismutase (N ligands) play important roles in mediating oxidative stress^2^: Cu,Zn superoxide dismutase catalyzes the disproportionation of superoxide radicals produced during oxygen metabolism^47^ while metallothionein is a chaperone protein involved in heavy metal detoxification^50^. In humans, expression of both proteins changes during carcinogenesis due to their respective roles in managing reactive oxygen species and Cu concentrations, which can rise during growth of cancerous tissue^51^. Consequently, an emerging body of research suggests a link between Cu isotopes and cancer^9,11,27–29,52^, and other diseases^20,21,53^.

Our findings support the hypothesis that changes in Cu isotope partitioning during cancer occur, in part, due to the expression of distinct Cu-binding biomolecules^9,27^. Serum δ^65^Cu is significantly lower in patients of colorectal and breast cancer^27^, as well as liver cancer (hepatocellular carcinoma)^9,26^ and cirrhosis^31^ relative to healthy individuals (− 0.26 ± 0.4‰, n = 49)^8^, with a proposed cutoff value of < − 0.35‰ indicating increased risk of mortality^27^. Several mechanisms have been hypothesized to explain these anomalies. Balter et al.^9^ ascribed low δ^65^Cu in the blood of hepatocellular carcinoma patients, which they related to increased concentrations, to release of metallothionein (S ligand)-bound Cu in endogenous stores. Conversely, Télouk et al.^27^ argued that shifts in serum δ^65^Cu of cancer patients occurs because lactate, which binds Cu^2+^ with hydroxyl (O) groups in cancer cells, accumulates ^65^Cu. Our experimental findings support the assumption underlying both hypotheses—that S ligands favor ^63^Cu and O ligands favor ^65^Cu. However, given the complexity of Cu trafficking and the likely importance of considerations apart from ligand identity (e.g., oxidation state, coordination geometry/ligand arrangement, metal abundance) additional work probing the relative importance of these variables is necessary to distinguish between them.

While recent work has focused on the medical potential of isotope metallomics, the work presented here is also relevant for using metal isotopes to investigate microbial metabolism in the environment. Though Cu has received less attention, several researchers have proposed biomarkers for application in the geologic record based on other transition metals, including Zn to track eukaryotes^54^, nickel (Ni) to trace methanogenesis^55^, and molybdenum (Mo) to investigate nitrogen assimilation pathways^56,57^. Metal isotopes are also increasingly being used to trace the sources, sinks, and cycling of metals in the ocean^58–60^. Unlike Zn^61–63^ and Ni^64^, marine primary producers do not appear to significantly fractionate the dissolved Cu pool^58^. Nevertheless, several studies have shown that prokaryotes can significantly fractionate Cu, with Δ^65^Cu_cells-media_ ranging from − 4.4‰^22^ to + 3.0‰^18^. Understanding why and when these biological fractionation effects occur in the environment will require further study, including consideration of when quantitative uptake may mute isotope fractionation. However, our work implicates changes in the Cu proteome of microbial communities as a potential factor driving differences in Cu isotope ratios.

## Conclusion

Here, we present the first experimental results characterizing the magnitude of Cu isotope fractionation during ligation by amino acids most commonly observed at protein metal-binding sites. Our work demonstrates that amino acids differentially fractionate Cu isotopes, with light ^63^Cu accumulating at the S sites of cysteine and heavy ^65^Cu accumulating at O sites of glutamate and aspartate. Our findings are in keeping with the few studies that have successfully measured Cu isotope ratios in in vivo proteins^17,34^, but differ in magnitude from computational estimates (Fig. 1)^4^, likely due to differences in coordination environment under modeled and laboratory conditions. This work supports the hypothesis that metalloprotein biosynthesis affects the distribution of transition metal isotopes within cells and suggests that the magnitude of computed isotope separation values should be interpreted with caution.

### Supplementary Information

Supplementary Information.

Supplementary Table 3.

Supplementary Table 4.

## Data Availability

The data presented here are available within the article and its online supplementary files.
