# Supplementary material for: Metal-binding amino acid ligands commonly found in metalloproteins differentially fractionate copper isotopes
Source: Sci Rep. 2024 Jan 22;14:1902. doi: 10.1038/s41598-024-52091-7 (PMC11229503; doi:10.1038/s41598-024-52091-7)
Supplement: Supplementary file 1 — Supplementary Information. [file 41598_2024_52091_MOESM1_ESM.docx]

Metal-binding amino acid ligands commonly found in metalloproteins differentially fractionate copper isotopes

Corday R. Selden^1,2^*, Kathrin Schilling^3^*, Linda Godfrey^1^, Nathan Yee^1,4^

^1^Department of Earth and Planetary Sciences, Rutgers University, Piscataway, NJ USA

^2^Department of Marine and Coastal Sciences, Rutgers, University, New Brunswick, NJ USA

^3^Department of Environmental Health Sciences, Mailman School of Public Health, Columbia University, New York City, NY USA

^4^Department of Environmental Sciences, Rutgers University, New Brunswick, NJ USA

*Corresponding authors: Corday R. Selden ([crselden@marine.rutgers.edu](mailto:crselden@marine.rutgers.edu)) and Kathrin Schilling ([ks3759@columbia.edu](mailto:ks3759@columbia.edu))


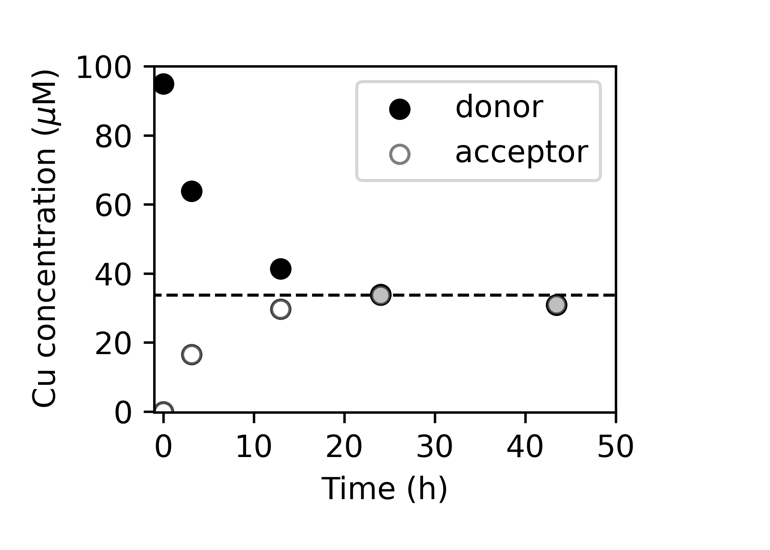


**Suppl. Fig. 1.** Time course of no-ligand (100% free Cu) control experiments. Equilibration of Cu between the acceptor (white) and donor (black) solutions shown as dots.


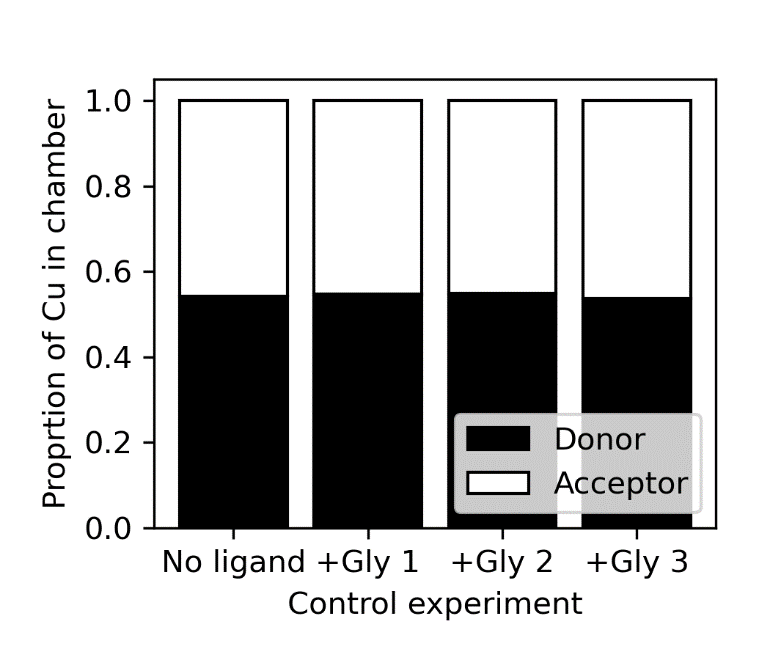


**Suppl. Fig. 2.** Distribution of Cu at t=final in three replicate experiments with 1:1 molar ratio of Cu to glycine (‘+Gly’) and a no-ligand control (‘No ligand’) which was run in parallel.

| Ligand | # replicate experiments | Initial  [AA]  (µM)^1^ | Initial [Cu]  (µM)^1^ | Final [Cu] (µM)^2^ | Proportion of Cu in donor chamber at t=f (%)^3^ | Bound Cu (µM)^4^ | Cu recovery following dialysis (%)^5^ |
| --- | --- | --- | --- | --- | --- | --- | --- |
| Cysteine | 3 | 53.2 | 114 | 85.5 ±2.8 | 83.4 ±0.4 | 66.9 ±0.8 | 70.5 ±2.7 |
| Cysteine | 3 | 52.5 | 121 | 89.6 ±5.7 | 78.5 ±3.0 | 57.1 ±6.0 | 78.9 ±5.8 |
| Histidine | 2 | 100 | 94.9 | 48.0 ±0.5 | 57.6 ±0.8 | 15.3 ±1.6 | 50.7 ±0.7 |
| Glutamate | 3 | 100 | 109 | 96.4 ±1.0 | 67.0 ±0.6 | 34.1 ±1.1 | 87.9 ±1.1 |
| Aspartate | 3 | 100 | 128 | 106 ±1 | 66.2 ±0.8 | 32.4 ±1.6 | 82.5 ±0.8 |

**Suppl. Table 1.** Conditions during dialysis of Cu-amino acid complexes (mean ±2SE).

^1^Initial solutions were the same for replicate experiments.

^2^Mean of the sum (acceptor + donor) of dissolved Cu in replicate dialyzers following dialysis per total volume

^3^Calculated as Cu mass in the donor at t=f divided by the sum Cu mass in the donor and acceptor chambers at t=f

^4^Bound Cu concentration was calculated as$\text{(1-ƒ}_{free})$ multiplied by the final Cu concentration; $\text{ƒ}_{free}$ is given in Eqn. 3. This approach assumes that only free Cu was lost to the membrane and that all amino acid was bound to Cu.

^5^The sum (acceptor + donor) of dissolved Cu in the system following dialysis as a percentage of the initial amount

| Solution | *This study*  δ^65^Cu_SRM976_  (‰)^1^ | *Literature value*  δ^65^Cu_SRM976_  (‰) | Reference |
| --- | --- | --- | --- |
| HICU-1 | -0.03 ±0.02 (n=20) | -0.04 ±0.03 (n=18) | Sullivan et al. 2020 |
| 1838 Cu penny | 0.05 ±0.02 (n=15) | 0.00 ±0.08 (n=NR)^2^ | Mathur et al. 2009 |
| Cu source solution | 0.89 ±0.02 (n=20) | N/A | N/A |
| Initial Cu-amino acid donor solutions^3^ | 0.88 ±0.07 (n=5) | N/A | N/A |

**Suppl. Table 2.** Cu isotope enrichment of standard reference materials (mean ±2SE).

^1^These values represent means across multiple instrument runs.

^2^Number of replicates not reported.

^3^This value represents the overall mean of the mean δ^65^Cu values (n=3) measured in the initial (pre-dialysis) Cu-amino acid solutions across all experiments (n=5). Error is propagated from 2σ for each solution.

**Suppl. Table 3.** Cu concentrations and δ^65^Cu values (mean ±2SE) for experimental solutions. Experimental conditions described in Section 2.2 of the main text.

**Suppl. Table 4.** Calculated results (Cu recovery, *f*_free_, δ^65^Cu_free_, δ^65^Cu_total_, δ^65^Cu_complexed_, Δ^65^Cu_complexed-free_) for individual experiments with amino acids. Pertinent equations given in Section 2.4 of the main text.
